# Life-Threatening Steroid-Refractory Colitis Following One Cycle of Neoadjuvant Immunotherapy and Chemotherapy for Early Triple-Negative Breast Cancer

**DOI:** 10.7759/cureus.95960

**Published:** 2025-11-02

**Authors:** Shahira Butt, Alice Rendall, Samantha Nimalasena, Uzma S Asghar

**Affiliations:** 1 Medical Oncology, The Royal Marsden NHS Foundation, London, GBR; 2 Radiation Oncology, Kent Oncology Centre, Maidstone and Tunbridge Wells NHS Trust, Maidstone, GBR; 3 Radiation Oncology, Breast Unit, The Royal Marsden NHS Foundation Trust, London, GBR; 4 Medical Oncology, Guys and St Thomas's NHS Foundation, London, GBR

**Keywords:** colitis, immunotherapy, infliximab, neoadjuvant chemotherapy, triple negative breast cancer

## Abstract

Triple-negative breast cancer (TNBC) is an aggressive subtype with a poor prognosis. Neoadjuvant chemotherapy combined with immunotherapy is the standard of care for stage 2-3 breast cancer. This case study describes the clinical history of one patient who experienced profound immune-related adverse events, specifically severe colitis, hepatitis and keratitis following one cycle of neoadjuvant chemotherapy and immunotherapy. The patient was hospitalised and then transferred to a tertiary cancer centre. She needed intensive treatment to treat the colitis, total parenteral nutrition (TPN), three different immunosuppressant drugs, antibiotics, antivirals, and supportive treatment. Breast surgery was performed whilst she was an inpatient, and she did not receive further systemic treatment. In order to avoid significant treatment-related morbidity, better biomarkers predicting immunotherapy toxicity and response are needed to help decide who will benefit from upfront surgery vs. neoadjuvant chemotherapy with immunotherapy.

## Introduction

Globally, one million women are diagnosed with breast cancer, and approximately 150,000 (10-15%) have triple-negative breast cancers (TNBC; estrogen receptor (ER) 0-2, progesterone receptor (PR) 0-2, human epidermal growth factor receptor 2 (HER2) 0-2) with 7,500 new cases of TNBC per year in England alone. TNBC has a poorer prognosis, with a higher number of women relapsing and a shorter life expectancy. Up to 50% of women diagnosed with stage 1-3 TNBC relapse, and approximately 40% of these women die within 5 years, despite treatment with curative intent. Consequently, a more aggressive treatment approach is taken. 

The KEYNOTE-522 study provides evidence that four chemotherapy drugs plus immunotherapy improves outcomes for women with stage 2-3 TNBC, and consequently, pembrolizumab plus neo/adjuvant chemotherapy has become the standard of care across several countries [1,2,3]. However, immunotherapy-related adverse drug reactions affect approximately 10% of participants in clinical trials. Immune-related adverse events can affect multiple organs, be permanent, and cause significant morbidity in a population with curable breast cancer expected to return to “normal” baseline [4]. 

We report a notable case of a patient hospitalised for 3.5 months following a single cycle of neoadjuvant immunotherapy plus chemotherapy for stage 2 TNBC, from which she never fully recovered due to this iatrogenic complication. Whilst immunotherapy is effective, it is also unpredictable, and further research is needed to understand the real-world impact of immune checkpoint inhibitors and biomarker research to help identify risk factors for developing severe immune-related toxicities.

## Case presentation

A 55-year-old female was diagnosed with stage 2 TNBC in November 2023. On imaging, she had a left-sided breast cancer measuring 40 mm on mammogram and 29 mm on breast ultrasound, with a normal axilla. The breast multidisciplinary team meeting decision was to treat with neoadjuvant chemotherapy and immunotherapy (pembrolizumab, carboplatin, paclitaxel, epirubicin, cyclophosphamide), followed by curative breast surgery. Her co-morbidities included resection of thymoma (pT1a Nx Mx, Nov 2021) and controlled schizophrenia, last needing hospitalisation over 4 years ago.

Nineteen days following administration of the first cycle of neoadjuvant chemotherapy and immunotherapy, she was hospitalised at her local district general hospital with grade 2 diarrhoea (grade 2 = increase of 4-6 stools/day over baseline), fever, a rise in liver function tests suggestive of liver injury, and visual disturbance, diagnosed as keratitis. She was started on IV antibiotics for infection, specifically tazoxin 4.5g QDS, IV methylprednisolone (1 mg/kg) for suspected immune checkpoint-induced (IO) colitis, topical steroids for the keratitis, and prophylactic co-trimoxazole. 

Her vision returned to normal within days. However, the colitis and hepatitis did not improve despite high-dose methylprednisolone (2 mg/kg). Therefore, on day five, the decision was made that this was steroid-refractory colitis (grade 3) and she was transferred to the tertiary cancer centre for consideration of infliximab. The reasons for hospital transfer were: the lack of a local patient management pathway enabling the Acute Oncology team or medical team to administer infliximab for a cancer patient, the concern that breast surgery for TNBC may need to be brought forward, and the concern that the next treatment cycle of neoadjuvant treatment may need to be given in a ward. At the time, none of these were possible at the local district general hospital, which had visiting oncologists only.

Investigations

**Figure 1:**
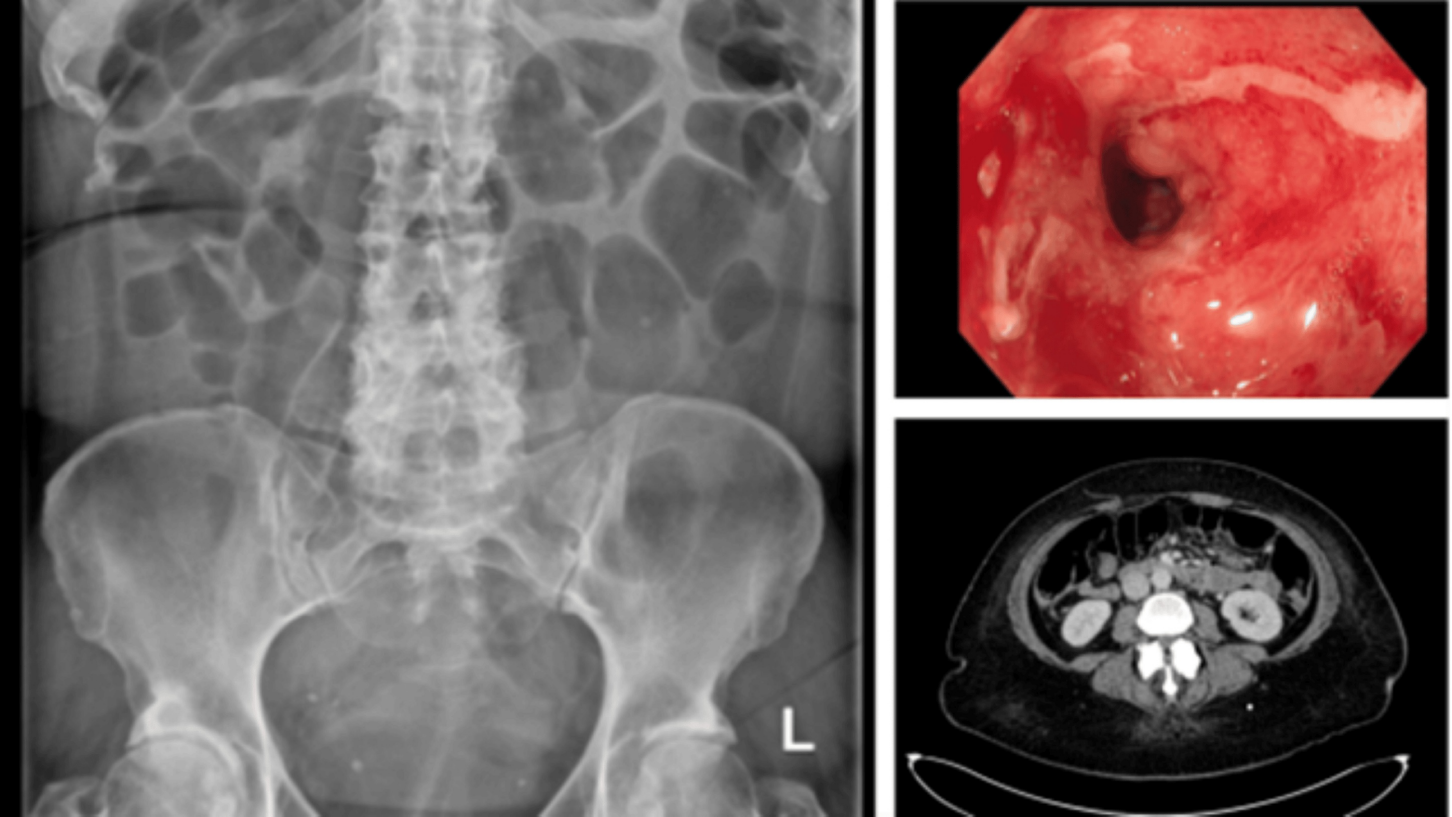
Abdominal X-ray and sigmoidoscopy Left: Plain abdominal X-ray demonstrating dilated bowel loops, specifically in the colon, suggestive of colitis.  Right-top:  Image from sigmoidoscopy confirming colitis. Right-bottom: CT image confirming colitis and the pattern of involvement.

Numerous investigations were conducted - from routine laboratory tests to specialist virology blood tests, faecal testing, imaging, colonoscopy (Figure 1), and liver biopsy. 

Hepatitis

**Figure 2:**
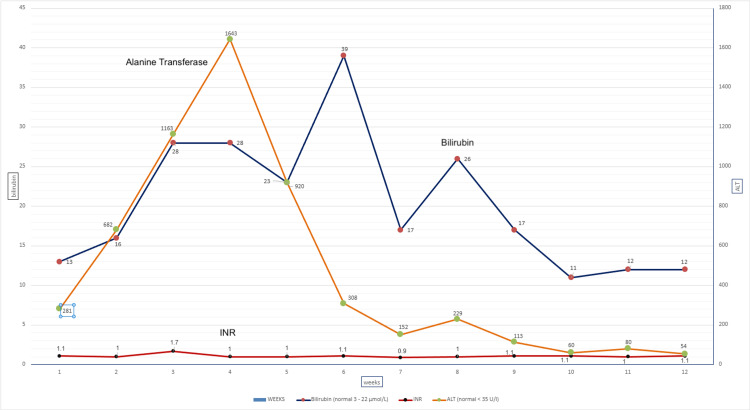
Liver function trend during the admission Fluctuations seen in alanine transferase (ALT), bilirubin, and international normalised ratio (INR) for clotting levels during the hospital visit. Normal ranges:  bilirubin 3-24 umol/L, ALT <35 U/L, INR <1.3 if not on anti-coagulation.

On admission, the alanine transferase (ALT) was abnormal and rising; hence, it was decided to initially start the immunosuppressant mycophenolate, 1 gram twice daily, as recommended by local Acute Oncology Service guidelines for pembrolizumab-induced hepatitis and colitis. The ALT levels steadily increased over 4 weeks to 1643 IU (46.94 x ULN; grade 4) despite mycophenolate (Figure 2). Viral serology was negative for Hepatitis A (IgM), Hepatitis B (Core Ab, Surface Ab), Hepatitis C, and HIV, and the autoimmune panel was negative for anti-mitochondrial antibody (AMA), anti-nuclear antibody (ANA), and liver-kidney microsomal antibodies (LKMA). The liver MRI was reported as normal; hence, an ultrasound-guided liver biopsy was performed by interventional radiology. The pathology report for the liver biopsy reported chronic inflammation of some portal tracts and parenchyma with evidence of interface hepatitis. 

Despite immunosuppressive treatment, liver function tests remained elevated (Grade 2 transaminitis), prompting a Magnetic Resonance Cholangiopancreatography (MRCP), which showed no further significant findings. The liver biopsy helped exclude autoimmune hepatitis and reinforced the decision to start infliximab treatment [5].

Colitis

The colitis was severe, occurring after the first cycle of neoadjuvant chemotherapy and immunotherapy for early TNBC. Prior to starting chemotherapy and immunotherapy, her bowel frequency was one to three times per day. On presentation to the local district general hospital, she had severe diarrhoea with watery stools with frank blood. She was opening her bowels more than 7x per day (grade 3, severe), and as a result was tachycardic, requiring medical intervention. Continuous intravenous hydration was needed to maintain stable blood pressure and adequate renal function over 3 months. Abdominal X-ray imaging (AXR) identified a dilated transverse colon (7 cm) with subtle thumbprinting consistent with acute colitis (Figure 1). CT abdomen highlighted that the colitis was localised to the distal descending colon, sigmoid colon, and rectum. Initial flexible sigmoidoscopy visually confirmed colitis with pseudomembranous formation occurring in the distal colon, although biopsy results were negative. A follow-up sigmoidoscopy on week 5 of the admission showed moderate to severe colitis involving the descending colon, sigmoid colon, and rectum, consistent with immune-related colitis (Figure 1) [6]. Stool testing for *Clostridium difficile *was negative.

Specialist review from gastroenterology and surgical teams highlighted concerns about the risks of perforation, and, if the colitis did not improve, she would need a pancolectomy; hence, her symptoms were closely monitored and she had regular monitoring with AXR, abdominal CT scans, faecal elastase quantification (pancreatic insufficiency) and faecal calprotectin (biomarker for intestinal inflammation). During this admission, four abdominal CT scans and seventeen AXRs were performed. There was no bowel perforation. Following a 3.5-month hospital admission, the diarrhoea improved significantly but never fully resolved.

**Figure 3:**
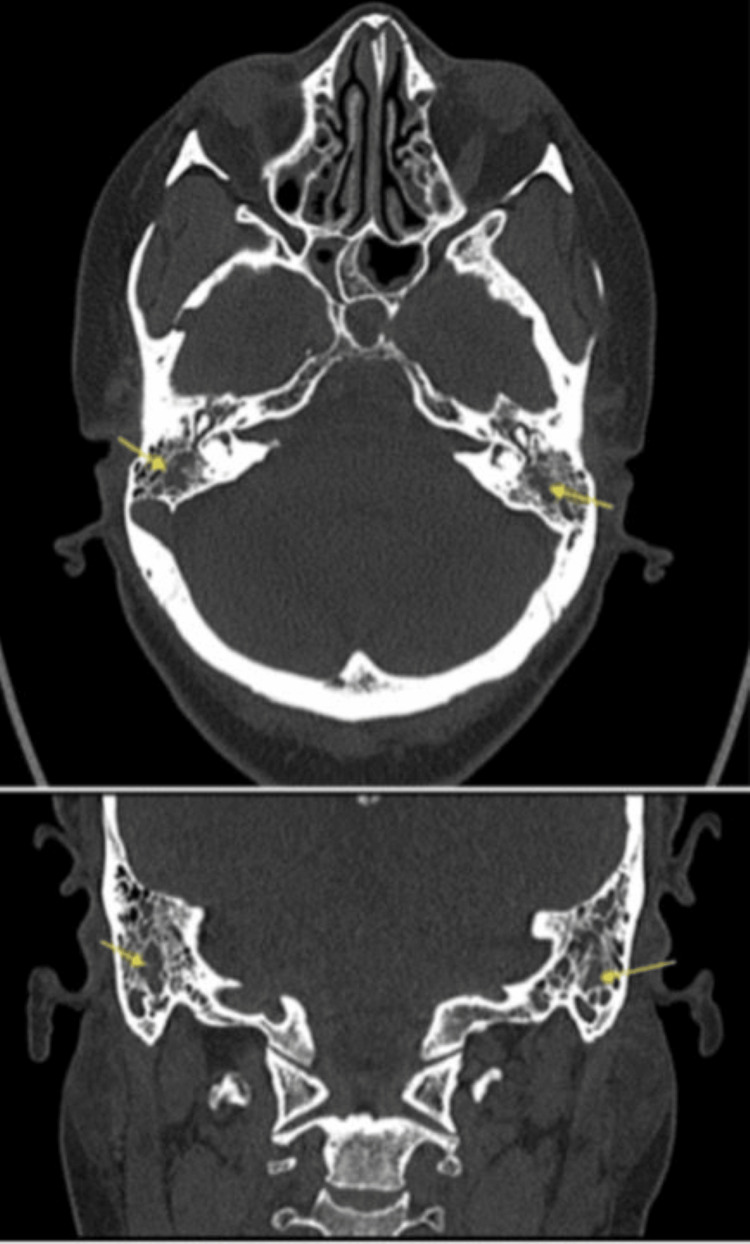
Bilateral mastoiditis The top image shows a cross-sectional view of CT Gead.  Arrows pointing at opacification (normally air) of bilateral mastoid air cells and no bony destruction, suggestive of bilateral mastoiditis. Similar changes are seen in the bottom image (arrows pointing at opacification in mastoid air cells).

This patient experienced intermittent fevers, requiring extensive infectious disease investigations, including blood cultures and site-specific cultures. During the fifth week, cultures taken from skin lesions in the natal clefts grew herpes simplex virus (HSV), requiring intravenous antivirals. In the tenth week, peripheral cultures grew vancomycin-resistant enterococcus (VRE) from the peripherally inserted central catheter (PICC) line and *Clostridium innocuum*, leading to targeted antimicrobial management. CT of the head and neck was also conducted for a suspected ear infection, which revealed bilateral mastoiditis, needing further antimicrobial therapy (Figure 3). 

The workup prior to the administration of immunosuppressive treatment included testing for tuberculosis (TB), blood tests for varicella zoster virus (VZV), cytomegalovirus (CMV), hepatitis and HIV, all of which were negative.  

Diagnoses

The main differential diagnoses for multi-organ involvement were immune-related adverse events (irAEs) following just one cycle of neoadjuvant chemotherapy, specifically colitis and immunotherapy-induced hepatitis.

Treatment

Stage 2 Triple Negative Breast Cancer Treatment

Initially, the planned management strategy was eight cycles of neoadjuvant chemotherapy and immunotherapy (21-day cycles) followed by breast-conserving surgery, radiotherapy, and adjuvant immunotherapy. The patient had received only one cycle of neoadjuvant intravenous carboplatin (AUC 1.5), paclitaxel (80 mg/m2 weekly) and pembrolizumab (200 mg) at the standard dose. Due to severe treatment-related toxicities and delayed recovery, further chemotherapy was not administered. This case was re-discussed at the breast multidisciplinary team meeting, and because it was challenging to treat the TNBC with further neoadjuvant chemotherapy, the breast surgery was brought forward to March 2024. 

She underwent left reduction mammoplasty and left sentinel lymph node biopsy whilst hospitalised because clinical examination suggested that the tumour in situ had increased in size, and CT imaging confirmed no distant metastases. The postoperative pathology report demonstrated invasive ductal carcinoma grade 3, measuring 50 mm in size, with high-grade ductal carcinoma in situ (DCIS). Lymphovascular invasion was not identified, and all margins were greater than 2 mm. Following surgery, she completed radiotherapy (26 Gy in five fractions to the left breast, followed by four out of a planned five fraction boost to the tumour bed (10.68/13.37Gy)). At her outpatient clinical, review both her doctors and the patient felt that further treatment with adjuvant pembrolizumab or adjuvant capecitabine was not in her best interests due to her ongoing diarrhoea and frailty. 

Immune Related Adverse Events (irAEs)

After Acute Oncology review, high-dose intravenous methylprednisolone was started at the local district general hospital initially at a dose of 1 mg/kg due to risks of bowel perforation and concerns about exacerbating the schizophrenia. After 24 hrs, the dose was increased to 2 mg/kg, reflecting recommendations by the UK Oncology Nursing Society (UKONS) guidelines for grade 3-4 colitis [7]. The bloody diarrhoea and rising levels of ALT continued despite >3 days of high-dose steroids, indicating steroid-refractory irAEs. The immunosuppressive treatment mycophenolate mofetil was started on day 5 according to local Acute Oncology Service (AOS) guidelines due to abnormal liver function tests (LFTs), and the steroid dose was gradually reduced and eventually stopped after 4 weeks, following a Synacthen test. Total parental nutrition (TPN) was administered for 3 weeks, due to a significant risk of malnutrition after 1 week of severe bloody diarrhoea and minimal oral intake. In week four of admission, infliximab (anti-tumour necrosis factor antibody; TNF) was administered twice, one week apart, which significantly accelerated recovery of both the colitis and hepatitis. 

Interestingly, the keratitis improved quickly with topical steroids and chloramphenicol eye drops during this period because her vision returned to baseline.

Infections

In order to minimise the risk of infections, prophylactic antifungals caspofungin and co-trimoxazole were given due to the high-dose immunosuppressive treatment. During this admission, she was treated for multiple infections, most likely due to prolonged immunosuppression. In week five of the admission, the patient developed skin lesions in the peri-anal region, which tested positive for herpes simplex virus (HSV) and received oral aciclovir 400 mg five times a day for 25 days. In week six, a CT of the head and neck area was conducted for suspected ear infection, which revealed bilateral mastoiditis, treated with tazocin and vancomycin antibiotics (Figure 3). In week ten, peripheral cultures grew vancomycin-resistant enterococcus (VRE) from the PICC line and clostridium innocuum, which was treated with tazocin, metronidazole, and linezolid antibiotics. 

This patient underwent curative breast surgery and was discharged after an inpatient stay of 3.5 months. She was unfortunately never well enough for further chemotherapy. Three months after breast surgery, a diagnosis of metastatic breast cancer was made, whilst being hospitalised at her local psychiatric unit due to a relapse of schizophrenia. Her treating physicians’ assessments were that she was too frail to attend outpatient oncology appointments and was reviewed via telephone consultations. She died 8 months after the breast cancer diagnosis due to metastatic breast cancer.

## Discussion

NICE guidelines [TA851] recommend adding pembrolizumab to neoadjuvant chemotherapy for early TNBC with a high recurrence risk to increase the chances of a pathological complete response, which is associated with a better prognosis [1,2,8]. This guidance is driven by the positive KEYNOTE-522 study which reported a 7.5% increase in pathological complete response with the addition of pembrolizumab, an improvement in disease free survival by 7.7% (HR 0.63, 95% CI 0.48-0.82, P < 0.001) [8] and an improvement in overall survival by 4.9% (P = 0.002) [9]. Consequently, immunotherapy with chemotherapy is standard practice in the NHS and in countries where pembrolizumab is accessible for stage 2-3 TNBC. However, there are no clear biomarkers predictive of immunotherapy response and no widely accepted biomarkers predictive of immunotherapy-associated toxicity. 

This is an unfortunate case where the treatment-related complications, specifically severe colitis and hepatitis, which were steroid refractory, resulted in significant morbidity with a detrimental impact on this lady's quality of life, an unexpected use of healthcare resources and difficulty in delivering planned cancer management. There are many case studies reporting pembrolizumab toxicities [9-11], some of which can be associated with improved outcomes or dramatic responses [10,11] but not in the early breast cancer space. If clinicians had access to robust tools for treatment stratification, especially important for early cancer, then possibly the treatment decision may have been different. Alternative management strategies could include upfront curative breast cancer surgery or neo/adjuvant chemotherapy alone, highlighting that although neoadjuvant immunotherapy plus chemotherapy has strong survival data, we do need to quantify the attrition rate resulting from drug toxicities causing significant delays or lost opportunities for breast surgery. 

## Conclusions

Neoadjuvant immunotherapy combined with chemotherapy can be associated with substantial morbidity, highlighting the need for real-world datasets to capture adverse events beyond the scope of clinical trials. Although these events were unexpected, improved systems are required for closer monitoring of patients receiving systemic anti-cancer therapy to enable rapid responses and early intervention. Clinicians should recognise that colitis may be steroid-refractory, and early use of infliximab, despite elevated liver function tests, may be preferable for effective symptom management.

In the early breast cancer setting, where patients have potentially curable disease, the threshold for treatment-related toxicity is considerably lower, emphasising the urgent need for biomarkers predictive not only of therapeutic response but also of drug-related toxicities. Emerging classes of agents, including immunotherapies and anti-TNF treatments, introduce novel and often unfamiliar toxicities in breast oncology, highlighting the importance of education for healthcare professionals. The management of immune-related adverse events extends beyond oncologists to include acute oncology teams and multidisciplinary specialists such as gastroenterologists, hepatologists, respiratory physicians, ophthalmologists, cardiologists, and endocrinologists, who are frequently involved in the care of hospitalised patients.
